# Antiretroviral Therapy Optimisation without Genotype Resistance Testing: A Perspective on Treatment History Based Models

**DOI:** 10.1371/journal.pone.0013753

**Published:** 2010-10-29

**Authors:** Mattia C. F. Prosperi, Michal Rosen-Zvi, André Altmann, Maurizio Zazzi, Simona Di Giambenedetto, Rolf Kaiser, Eugen Schülter, Daniel Struck, Peter Sloot, David A. van de Vijver, Anne-Mieke Vandamme, Anders Sönnerborg, for the EuResist and Virolab study groups

**Affiliations:** 1 Clinic of Infectious Diseases, Catholic University of Sacred Heart, Rome, Italy; 2 Informa CRO, Rome, Italy; 3 College of Medicine, Pathology, Immunology and Laboratory Medicine, EPI, University of Florida, Gainesville, Florida, United States of America; 4 IBM Haifa Research Lab, Haifa, Israel; 5 Max Planck Institute for Informatics, Saarbrücken, Germany; 6 University of Siena, Siena, Italy; 7 University of Cologne, Cologne, Germany; 8 Centre de Recherche Public-Santé, Luxembourg, Luxembourg; 9 Universiteit van Amsterdam, Amsterdam, The Netherlands; 10 Department of Virology, Erasmus MC, University Medical Centre Rotterdam, Utrecht, The Netherlands; 11 Rega Institute, Katholieke Universiteit Leuven, Leuven, Belgium; 12 Karolinska Institute, Stockholm, Sweden; BC Centre for Excellence in HIV/AIDS, Canada

## Abstract

**Background:**

Although genotypic resistance testing (GRT) is recommended to guide combination antiretroviral therapy (cART), funding and/or facilities to perform GRT may not be available in low to middle income countries. Since treatment history (TH) impacts response to subsequent therapy, we investigated a set of statistical learning models to optimise cART in the absence of GRT information.

**Methods and Findings:**

The EuResist database was used to extract 8-week and 24-week treatment change episodes (TCE) with GRT and additional clinical, demographic and TH information. Random Forest (RF) classification was used to predict 8- and 24-week success, defined as undetectable HIV-1 RNA, comparing nested models including (i) GRT+TH and (ii) TH without GRT, using multiple cross-validation and area under the receiver operating characteristic curve (AUC). Virological success was achieved in 68.2% and 68.0% of TCE at 8- and 24-weeks (n = 2,831 and 2,579), respectively. RF (i) and (ii) showed comparable performances, with an average (st.dev.) AUC 0.77 (0.031) vs. 0.757 (0.035) at 8-weeks, 0.834 (0.027) vs. 0.821 (0.025) at 24-weeks. Sensitivity analyses, carried out on a data subset that included antiretroviral regimens commonly used in low to middle income countries, confirmed our findings. Training on subtype B and validation on non-B isolates resulted in a decline of performance for models (i) and (ii).

**Conclusions:**

Treatment history-based RF prediction models are comparable to GRT-based for classification of virological outcome. These results may be relevant for therapy optimisation in areas where availability of GRT is limited. Further investigations are required in order to account for different demographics, subtypes and different therapy switching strategies.

## Introduction

Current HIV-1 guidelines recommend genotypic resistance testing (GRT) both before starting antiretroviral therapy (ART) and at treatment failure. However, appropriate funding and/or facilities to perform GRT may not be available in low to middle income countries (LMIC), leaving physicians with switching therapy based solely on the patient's clinical background. Coupled with virological and immunological measurements, treatment history (TH) is one of the most crucial factors to play a role in the response to a new treatment regimen. In fact, current (2010) recommendations of the International AIDS Society-USA Panel [1] state that treatment history should be considered when designing a new regimen.

In LMIC there is still need for cheap viral load tests to identify early viral failures and limit the emergence of resistance [2]. In the past five years there has been an increase in HIV/AIDS surveillance, but there is still a general lack of rational collection and utilisation of the data as well as of appropriately trained medical staff [3], [4], [5]. Specific approaches for ART management have been designed by the World Health Organization [6]. Public health programs leading to earlier HIV diagnosis and initiation of ART are expected to improve patient outcomes in LMIC [7].

It has been shown that drug costs are a major factor impairing optimal HIV-1 drug sequencing [8]. Access to second-line ART regimens in LMIC is problematic, mainly because of the expense of HIV protease inhibitors (PI) [9], and CD4 monitoring is often the sole surrogate marker available to guide the management of the infection [10]. However, immunologic criteria to predict which patients have not achieved virological suppression results in significant misclassification of treatment responses [11]. Attempts to model the virological efficacy of ART in LMIC have been proposed by Colebunders et al. [12].

Given the need for low-cost viral load assays, evaluations of alternative technologies have been carried out [13], [14]. The costs for genotype resistance testing (GRT) also remain prohibitively high for most LMIC, with only a few facilities being able to support the expenses, execute the tests and exploit the results [15]. New relatively cheap technologies for GRT and non-B subtypes analysis have also been proposed [16], but these have not yet been propagated sufficiently. An additional challenge is the genetic diversity of HIV-1, since most infected patients in developing areas harbour non-B subtypes that could respond to therapy differently from subtype B, the clade which has been extensively treated in Western countries [17], [18].

The virological benefit of genotype-guided treatment decisions has been convincingly demonstrated in the last decade [19], [20], [21]. Although investigated to a lesser extent, genotype-guided treatment decisions were shown to be superior to those based on the medical history [22]. Accordingly, there is a plethora of GRT-based decision systems either for single drug susceptibility scoring or combined ART (cART) optimisation, from rule-based [23], [24], [25] to machine learning approaches [26], [27], [28], [29], [30], [31], [32]. On the other hand, attempts to guide treatment decisions using the TH information alone have been exploited only recently, using artificial neural networks, with extremely promising results [33].

In this work we aimed at investigating a set of statistical learning models – called random forests - for optimisation of antiretroviral therapy in the absence of GRT information, using the treatment history as a surrogate predictor. Furthermore, we compare the performance of TH-based to those of GRT-based models.

## Methods

### Ethics statements

This study uses anonymous retrospective data from European merged study cohorts and biological material was not employed in any step of the analysis. All the single data providers had previously obtained patients' informed consent for the execution of retrospective studies and their inclusion in merged cohorts, accomplishing national and international ethical issues. The study design was eventually approved by a global scientific committee and by local scientific committees of each single data provider.

### Study design

The EuResist retrospective database (http://www.euresist.org/) was used to extract patients' treatment change episodes (TCE), previously introduced by the HIV Resistance Response Database Initiative [31] and by the Forum for Collaborative HIV Research [34], in the so-called form of standard datum (SD).

Each SD was defined as a patient's new regimen (TCE), either a first-line or a subsequent line of therapy, coupled with a follow-up HIV-RNA measured at 8 weeks (ranging from 4 to 12) and at 24 weeks (ranging from 20 to 28) of unmodified therapy.

TCE included antiretroviral compounds approved by the Food and Drug Administration and by the European Medicine Agency: the nucleoside/nucleotide reverse transcriptase inhibitors (NRTI) lamivudine, abacavir, zidovudine, stavudine, zalcitabine, didanosine, emtricitabine and tenofovir disoproxil fumarate; the non-NRTIs (NNRTI) efavirenz, nevirapine and etravirine; the protease inhibitors (PI) amprenavir/fosamprenavir, atazanavir, indinavir, lopinavir, nelfnavir, full-dose ritonavir, boosting-dose ritonavir, saquinavir, tipranavir and darunavir; the fusion inhibitor enfuvirtide. Each drug was represented by a binary variable encoding its presence/absence in a TCE. No restrictions concerning the number of drugs included in a regimen were applied. Both suboptimal treatment regimens made of <3 drugs or salvage regimes with >4 drugs were included. It was previously shown that the inclusion of suboptimal TCE can improve data-driven model performance [31]. The number of drugs in the regimen was considered as a numerical variable.

Each TCE was provided with a contemporary HIV-1 genotype (spanning protease and reverse transcriptase genes) or the closest within 90 days before the TCE start date. The viral genotype was encoded as binary vector of mutations, insertions and deletions with respect to the HIV-1 consensus B reference, and the viral subtype was determined by a BLAST search on the latest subtype reference set provided by the Los Alamos repository (http://www.hiv.lanl.gov/).

The closest (e.g. baseline) HIV-RNA and CD4+/CD4% cell count measurements previous to the TCE start date (obtained at most 90 days before, provided that no other therapies were started and stopped during this time window) were collected. Patient's demographic information was also retrieved, including age, gender, mode of HIV transmission (drug user, heterosexual, homosexual/bisexual, blood products, mother-to-child transmission), and country of origin.

We associated to each TCE the previous patient's drug exposures, codifying a binary variable for each compound if that drug was experienced for more than 12 months. Three additional binary variables were defined summarising the exposures to the NRTI/NNRTI/PI classes, and a numerical variable representing the regimen line (any drug change in a combination therapy for any reason).

We defined a virological success at 8-weeks, as the achievement of an undetectable HIV-RNA or a decrease of at least 2 Log_10_ from the baseline HIV-RNA. At 24-weeks, the virological success was defined as the achievement of an undetectable viral load. Because of the inclusion of many viral load data obtained with non-ultrasensitive assays, a 500 cp/ml threshold was applied for the definition of undetectable HIV-RNA.

Additional details for the study design, the SD definition and data extraction procedures and constraints have been discussed previously [30], [31], [34]. For simplicity, we will refer to the two defined 8-week and 24-week SD sets, containing viral genotypic information, as SD8G and SD24G, respectively. Of note, these TCE correspond exactly to those used in [30], in order to maintain a fair comparison with other tested methods, such as expert rule bases. Indeed, the EuResist DB is periodically updated from the local sources (currently Italy, Germany, Sweden, and Luxembourg) participating to the network.

Two other data sets were extracted using the same notion of SD, except for relaxing the need of a baseline GRT, and including additional data from the Virolab study group (http://www.virolab.org/), comprising the cohorts of Belgium (Katholieke Universiteit Leuven) and Spain (fundaciò irsiCaixa). These two sets, containing treatment history but not mandatorily baseline GRT, were named SD8H and SD24H, respectively.

### Statistical methods

Random Forests (RF) were used to predict 8- and 24-week outcomes and to assess variable importance [35]. RF are a non-linear statistical learning methodology for classification and regression. They ensemble several decision trees (usually from hundreds to thousands) and give predictions by taking either the majority vote or the average of the single trees' outputs. Specifically, each single tree is fully grown on a bootstrap sample of the training data, without pruning the leaves, and at each node split only a subset of the variables is considered. RF present many advantages with respect to other non-linear machine learning methods, such as neural networks, since they usually yield high performance, are robust to over-fitting, can handle a large number of variables, and provide a measure of variable importance.

In our study, the tree number and number of candidate variables at each split of RF were optimised preliminarily with a bootstrap approach. The area under the receiver operating characteristic curve (AUC) [36] was adopted to evaluate the model fit. The AUC is equal to the probability that a classifier will rank a randomly chosen positive instance higher than a randomly chosen negative one, whereas a receiver operating characteristic (ROC) plot provides a graphical evaluation of true-positives (sensitivity) versus false-positives (1-specificity) tradeoffs. Multiple 10-fold Cross Validation (CV) and Bengio's corrected t-test [37], [38], controlling the false discovery rate with Benjamini-Hochberg method, were used to compare model performance.

The following nested models were defined: (i) full set of input covariates (including GRT and TH); (ii) full set of input covariates, excluding GRT information, but keeping TH; (iii) full set of input covariates, excluding TH, but keeping GRT; (iv) current cART, baseline HIV-1 RNA and CD4; (v) current cART.

We also designed a set of sensitivity analyses as follows: in order to investigate the potential bias derived from the fact that most of the TCE present in the data base have been decided after a GRT, we excluded from SD8H/SD24H all TCEs for which a baseline GRT was available, and we compared models with/without baseline HIV-RNA load as a covariate. From this reduced data set, we selected first- and second-line regimens commonly available in LMIC (TCE containing enfuvirtide, tipranavir, darunavir, and etravirine were removed) and repeated model evaluations. Finally, SD8G was split into two sets according to the viral subtype of each associated GRT, grouping B and non-B subtypes. RF models were trained on the subtype B dataset and tested against the non-B.

The analyses were carried out using *R*, open-source software for statistical computing [39], and *Weka*, a data-mining suite [40].

## Results

### Study population

From the EuResist data base, we collected 2,831 and 2,579 standard datum instances for the 8-week (SD8G) and 24-week (SD24G) outcome, respectively. Virological success was reported in 68.2% and 68.0% of cases, respectively. Suboptimal therapies (dual- and mono-therapies), had a prevalence of 6.2% and 5.5% in the 8-week and 24-week data set. The majority of cART contained lamivudine (55.9%), any PI/r (42.7%, where lopinavir/r accounted for 36.2%), tenofovir (34.8%), zidovudine (31.7%), didanosine (28.4%), stavudine (24.0%), efavirenz (17.5%), abacavir (16.0%), and nevirapine (10.6%). Darunavir, tipranavir, etravirine, enfuvirtide, raltegravir, and maraviroc were not present in the data set. Additional details on cART distribution are available in [30] or can be provided by posting a request to the EuResist study group.

Relaxing the need for a baseline GRT, the data sets SD8H and SD24H were extracted (n = 12,932 for both outcome points). Seventy-one percent of patients reached virological success at 8-weeks and 67% of patients reached virological success at 24-weeks. Differences in proportions of SD8H and SD24H with SD8G and SD24G success rates yielded p<0.0001 and p = 0.13, respectively. The percentage of suboptimal therapies was 8%. The majority of cART contained lamivudine (63%), zidovudine (33%), tenofovir (28%), stavudine (27%), any PI/r (29%, where lopinavir/r accounted for 23%), didanosine (21%), abacavir (19%), and efavirenz (11%). There was a low percentage of darunavir (1.3%) and tipranavir (1.3%) containing cART.

Table 1 summarises patients' baseline characteristics both for SD8G and SD8H.

**Table 1:** Patients' baseline characteristics.

Factor	SD8G	SD24G	SD8H/SD24H
Average (SD) patient age years	42 (13)	42 (13)	46 (9) *
Male gender	70%	70%	72% *
Mode of HIV-1 transmission			
Intravenous drug users	27%	27%	21% *
Homosexual men	32%	35% *	33% *
Heterosexual	38%	35% *	30% *
Nationality			
European or North American	72%	73%	71%
Previous exposure to antiretroviral classes (> = 1 year)			
NRTI	74%	71% *	59% *
NNRTI	41%	40%	24% *
PI	58%	55% *	43% *
Median (IQR) number of previous treatment lines	3 (1−6)	3 (0−6) *	3 (1−7) *
Median (IQR) number of drugs included in the cART	3 (3−4)	3 (3−4)	3 (3−4) *
Laboratory markers			
Median (IQR) HIV-1 RNA load Log_10_ cp/ml	4.4 (3.8−5.0)	4.4 (3.7−5.0)	4.0 (2.2−4.9) *
Median (IQR) CD4+ count cells/mm^3^	255 (137−397)	276 (152−384)	285 (160−449) *
Subtype distribution			
B	83%	85% *	n/a
C	3%	3%	n/a
02_AG	2.5%	2.2%	n/a
F1	2.3%	1.7%	n/a
Resistance mutations			
Median (IQR) no. of IAS NRTI mutations	1 (0−3)	1 (0−3)	n/a
Median (IQR) no. of IAS NNRTI mutations	0 (0−1)	0 (0−1)	n/a
Median (IQR) no. of IAS PI mutations	3 (2−5)	3 (2−5)	n/a

Summary of patients' baseline characteristics for 8- and 24-weeks data sets with a baseline GRT available (SD8G, n = 2,831; SD24G, n = 2,579) and the data set without a baseline GRT (SD8H and SD24H, n = 9,623). Values with * highlight significant differences between SD8G and SD24G or between SD8G and SD8H (p<0.05, by t-test, Wilcoxon rank sum or differences in proportion where appropriate).

### Comparison between GRT-based and TH-based RF models

**Figure 1:**
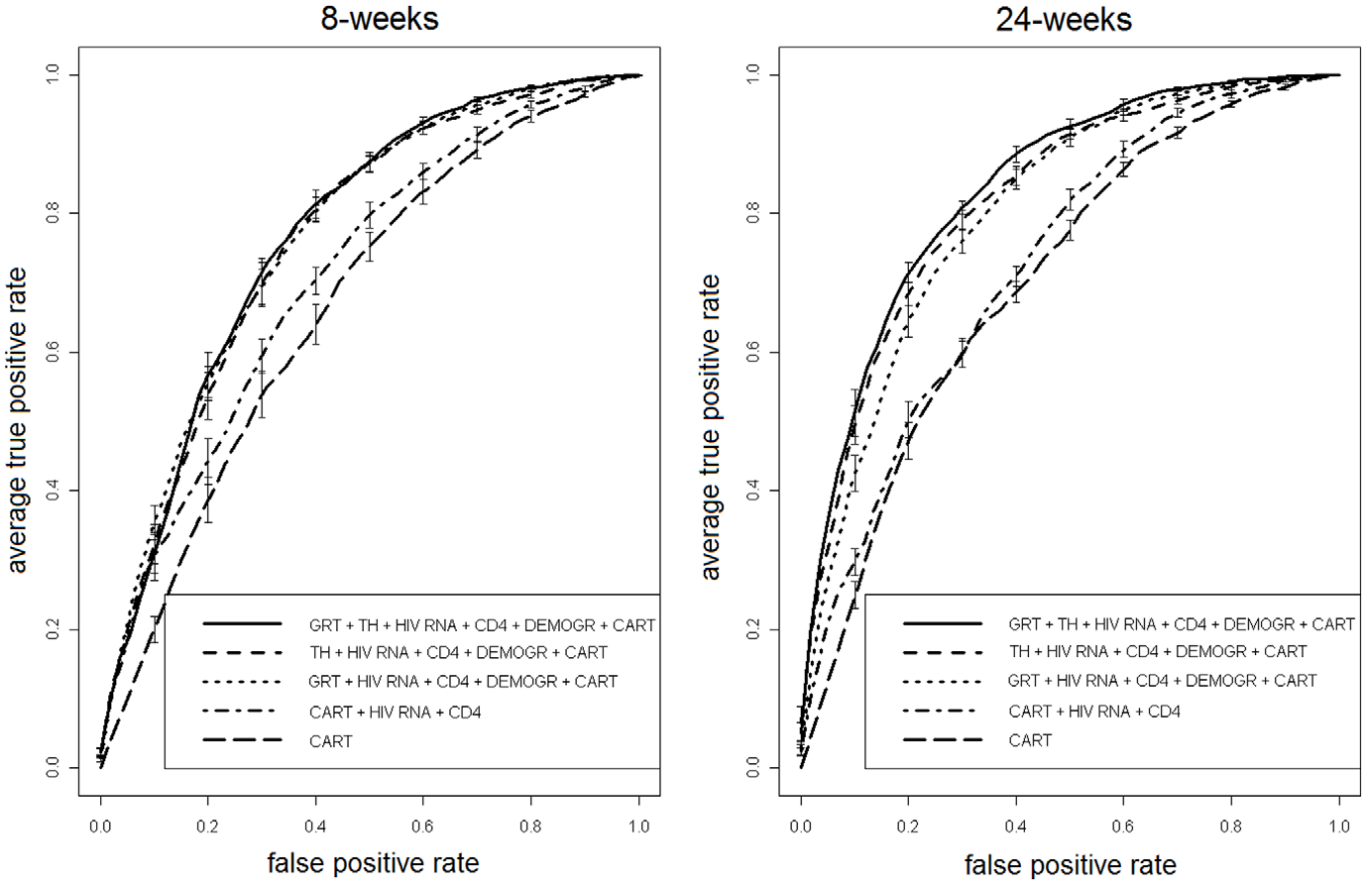
ROC analysis of models' performance. ROC plots of a single 10-fold CV run for RF models (i) through (v) for 8- and 24-weeks outcome (SD8G and SD24G).

Table 2 and Figure 1 show detailed performance results and ROC plots for SD8G and SD24G, comparing RF models (i) through (v), under multiple 10-fold CV. Model (i), which includes the full set of covariates (including both GRT and TH), was the best performing in terms of AUC for both time points. However, model (ii) (TH but not GRT) had AUC distributions not significantly different from model (i) at 8-weeks (p = 0.25) although inferior at 24-weeks (p = 0.04). The same held for model (iii) (GRT but not TH), which was comparable to (i) at 8-weeks (p = 0.28) and inferior at 24-weeks (p<0.0001). On the other hand, models (iv) and (v) were always significantly outperformed by models (i) to (iii) at any time point (all p<0.0001). Model (ii) and (iii) did not show significant differences in AUC at both time outcomes (p = 0.7 and 0.113).

**Table 2:** Model performance.

RF model	input variable set	10×10-fold CV AUC	
		8-weeks outcome (SD8G)	24-weeks outcome (SD24G)
		average (st.dev.)	average (st.dev.)
(i)	GRT + TH + HIV RNA + CD4 + DEMOGRAPHIC + cART	0.77 (0.031)	0.834 (0.027)
(ii)	TH + HIV RNA + CD4 + DEMOGRAPHIC + cART	0.757 (0.032)	0.821 (0.025)
(iii)	GRT + HIV RNA + CD4 + DEMOGRAPHIC + cART	0.762 (0.035)	0.807 (0.025)
(iv)	cART + HIV RNA +CD4	0.699 (0.037)	0.72 (0.03)
(v)	cART	0.65 (0.041)	0.687 (0.03)

Summary of area under the receiving operating characteristic curve (AUC) values for RF trained with selected input variable sets, calculated over ten multiple runs of 10-fold cross-validation (8-weeks and 24-weeks outcome).

GRT  =  genotype resistance test.

TH  =  treatment history.

cART  =  combination antiretroviral therapy.

**Figure 2:**
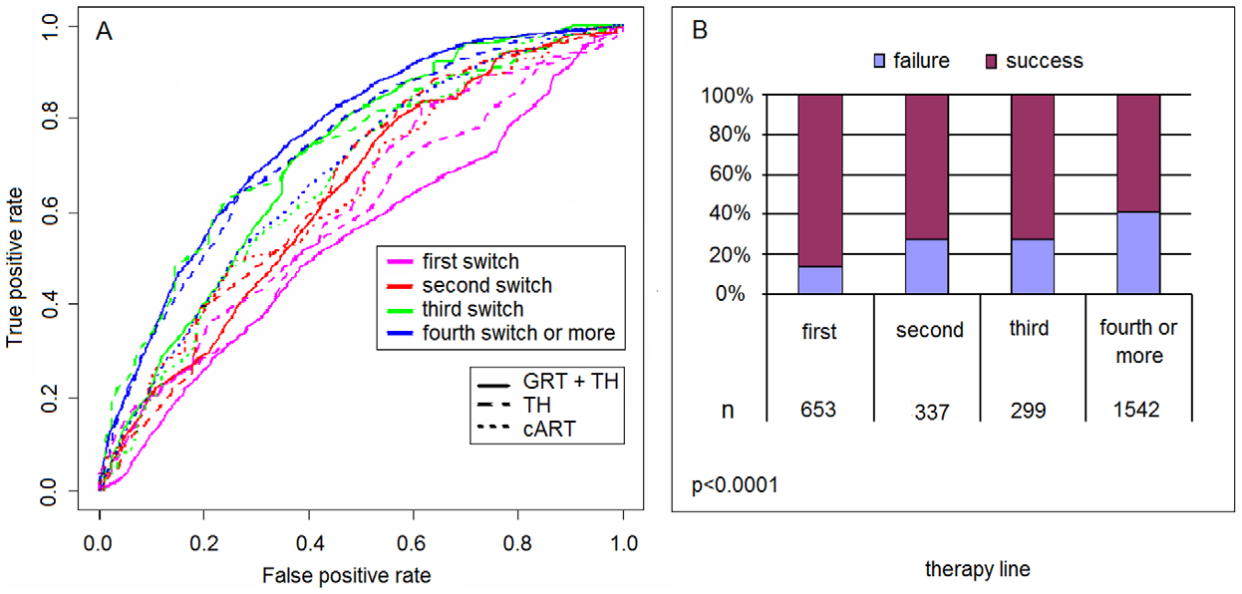
Models' performance evaluation by therapy line. Plot (*panel A*) of AUC of 10-fold CV for models (i), (ii) and (iv) by stratifying for therapy line (SD8G). Performance decrease significantly by decreasing the number of drug switches, and the loss in AUC is as pronounced in TH-based as in the other models. The proportion of virological successes (*panel B*) decreases significantly by increasing the therapy line (p<0.0001).

We also compared AUC of models (i), (ii), and (v) by stratifying for the therapy line (first-, second-, third-, fourth-line or more). Detailed results are shown in Table 3. Interestingly, both model (i) and (ii) show significantly better performance as compared to the base cART model (v) only at late switches. Figure 2 (panel a) depicts the AUC for each model and therapy strata over a single 10-fold CV run using the SD8G data set, and the proportion of virological successes for each therapy line (panel b).

**Table 3:** Model performance by therapy line.

RF model	input variable set	10×10 fold average AUC (st.dev.)							
		8-weeks out come (SD8G)				24-weeks out come (SD24G)			
		first-line	second-line	third-line	fourth-line or more	first-line	second-line	third-line	fourth-line or more
		n = 653	n = 337	n = 299	n = 1542	n = 671	n = 314	n = 254	n = 1340
(i)	GRT + TH + HIV RNA + CD4 + DEMOGRAPHIC + cART	0.56 (0.09)	0.60 (0.11)	0.71 (0.11)	0.78 (0.04)	0.61 (0.17)	0.64 (0.12)	0.68 (0.12)	0.80 (0.03)
(ii)	TH + HIV RNA + CD4 + DEMOGRAPHIC + cART	0.62 (0.10)	0.62 (0.11)	0.66 (0.11)	0.75 (0.03) *	0.59 (0.16)	0.63 (0.12)	0.68 (0.11)	0.79 (0.04)
(v)	cART	0.58 (0.11)	0.53 (0.10)	0.58 (0.11)*	0.62 (0.05) *	0.55 (0.17)	0.64 (0.09)	0.60 (0.12)	0.61 (0.05) *

Summary of area under the receiving operating characteristic curve (AUC) values for RF trained with selected input variable sets, calculated over ten multiple runs of 10-fold cross-validation (8-weeks and 24-weeks outcome), by stratifying for therapy line.

*p<0.05 with respect to the best model.

GRT  =  genotype resistance test.

TH  =  treatment history.

cART  =  combination antiretroviral therapy.

When executing CV on SD8H and SD24H, only RF models (ii), (iv) and (v) were testable (since GRT information was not present). Average (st.dev.) AUC values for model (ii) were 0.799 (0.011) at 8-weeks, and 0.832 (0.009) at 24 weeks. Model (iv) had average (st.dev.) AUC of 0.742 (0.012) at 8-weeks, and 0.752 (0.012) at 24 weeks. Model (v) had an average AUC of 0.684 (0.014) and 0.714 (0.013) at 8- and 24-weeks, respectively. Differences in AUC between (ii) and (iv) were significant both at 8-weeks and at 24-weeks (p<0.0001). As expected, the difference was even larger between model (ii) and (v) (p<0.0001 both at 8- and 24-weeks).

**Figure 3:**
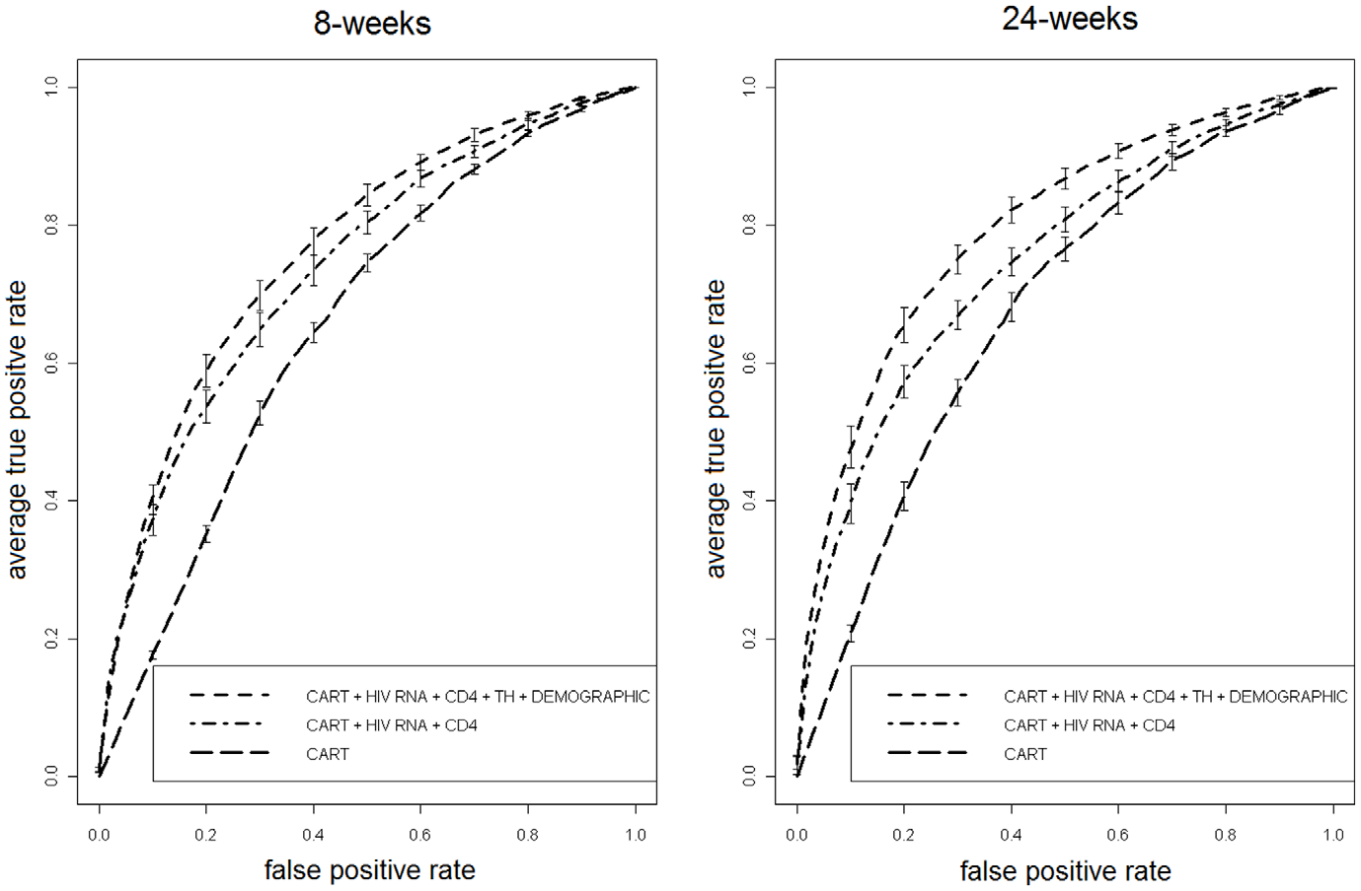
ROC analysis of GRT-free model performance. ROC plot of a single 10-fold CV run for RF models (ii) vs. (iv) vs. (v) for 8- and 24-weeks outcome (SD8H and SD24H).

Figure 3 depicts ROC curves both for SD8H and SD24H.

### Sensitivity analyses

Since all the extracted data sets may be biased due to the fact that in Europe the prevalence of genotype-guided TCE is high, we excluded from SD8H and SD24H all the TCE for which there was a baseline GRT available (obtaining n = 9,623). This is meant to be an approximation to the set of non-genotype-guided therapies, although some baseline GRT may have been available but not inserted in the data base. In a sub-analysis, then, we deleted the HIV-RNA variable. The proportion of successes was 71% and 65% at 8- and 24-weeks, respectively. For the 8-weeks outcome, average (st.dev.) AUC for models (ii), (iv), and (v) were 0.809 (0.015), 0.753 (0.015), and 0.685 (0.017). For the 24-weeks outcome, average (st.dev.) AUC for model (ii), (iv), and (v) were 0.839 (0.013), 0.765 (0.014), and 0.709 (0.018). Model (ii) significantly outperformed both models (iv) and (v) at all time points (p<0.0001). When removing the HIV-RNA variable, for 8-weeks outcome the average (st.dev.) AUC for models (ii) and (iv) were 0.758 (0.015) and 0.654 (0.016), p<0.0001; for 24-weeks outcome, the average (st.dev.) AUC for models (ii) and (iv) were 0.807 (0.015) and 0.670 (0.014), p<0.0001. Notably, model (ii) without baseline HIV-RNA outperformed model (iv) with baseline HIV-RNA at 24-weeks (p<0.0001), but not at 8-weeks (p = 0.4387).

Models (ii), (iv) and (v) were also tested on SD8H and SD24H excluding not only GRT-guided TCE, but also restricting to first- (NRTI/NNRTI) and second-line (NRTI/NNRTI/PI) regimens (n = 3,640) available in LMIC. Percentage of virological successes at 8- and 24-weeks were 79% and 75%. For the 8-weeks outcome, model (ii) had an average (st.dev) AUC of 0.76 (0.03), significantly higher (p<0.0001) than model (iv) and (v), that yielded AUC of 0.71 (0.03) and 0.70 (0.03), respectively. The same held for the 24-weeks outcome, where model (ii) had an average (st.dev) AUC of 0.81 (0.02), significantly higher (p<0.0001) than model (iv) and (v), with AUC of 0.75 (0.03) and 0.74 (0.03), respectively.

As a final evaluation, a special training/test data split was used for SD8G. The split was based on the subtype of the virus. Precisely, TCEs were grouped into subtype B or non-B, resulting in n = 2,353 and 478 instances, respectively. RF model (i) and (ii) were trained and 10-fold cross-validated on the B subtype set, and then tested against the non-B set. The proportion of successes at 8-weeks was 67% for B and 73% for non-B subtypes. In a single 10-fold CV run, model (i) yielded an average AUC of 0.765, model (ii) of 0.757, and model (iii) of 0.753. On the test set (i.e. non-B subtypes), AUC was 0.721 for model (i), 0.721 for model (ii), and 0.653 for model (iii).

AUC plots for the sensitivity analyses, along with variable importance evaluation on the SD8H data set, are available as supplementary material (Figure S1, Figure S2 and Material S1).

## Discussion

Following the demonstration of a major role for HIV-1 drug resistance mutations in response to antiretroviral therapy, GRT has been the standard decision tool to face HIV-1 drug resistance in clinical practice. The caregiver can use one or multiple systems developed to translate HIV-1 genotype into clinically relevant information. Commonly used genotype interpretation systems do not integrate additional patient information as an input to complement HIV-1 genotype in the effort to predict response to cART. A few experimental systems derived from statistical learning have recently been described which integrate patient and virus information to build an effective antiretroviral regimen [30], [31], [41]. These systems confirmed that TH has an impact on prediction of response to therapy.

The expanding cART coverage of LMIC poses new challenges for optimising treatment due to limited availability of second-line and later regimens and frequent lack of facilities or funding to support GRT. Our analysis suggests that availability of basic patient data supplemented with simple categorical indicators of past treatment can provide short- and medium-term response prediction as accurately as in the presence of HIV-1 genotype information. This finding is not surprising since the virus genotype is actually shaped by anti-HIV-1 compounds and can thus summarize the patient TH. It is interesting to note that the performance of the GRT-based model, which did not include any information on TH, decreased, although not significantly, at 24-weeks, compared to that of TH-containing models. This may be a consequence of the fact that bulk genotyping does not capture minority variants that have been selected by previous exposures [42] or resistant variants that survived in a reservoir and will be quickly reselected. It should be worth evaluating whether using the cumulative or historical genotype, made by the sum of all the available genotypes, improves accuracy at a later time point [43], [44]. Application of ultra-deep sequencing procedures could also provide high-sensitivity genotype data [45] possibly resulting in better prediction of response to therapy. We also found that both the RF models with GRT+TH and the TH alone increase their performance in predicting the virological outcomes when considering subsequent therapy lines. This suggests that the outcomes of the first/second lines are somehow less dependent on the viral strain or on the early ART exposures, while the evaluation of TH and the execution of a GRT boost up the confidence in predicting the correct outcome at later ART stages. In principle, continuous measures of exposure to therapy, i.e. corrections based on duration and last time of use, could improve the power of TH as a covariate. However, such detailed information is often hard to obtain, particularly in the absence of centralised (electronic) medical records. The simplicity of the input information should expedite further training of TH-based systems with data derived from these areas. In this context, the data required for querying a TH-based expert system should be kept simple in order to encourage its use. The input dataset investigated in this work was made of demographic data typically available at any HIV clinic (patient age, gender and route of infection), baseline markers (CD4 cell count and HIV-RNA load), just coupled with binary indicators for past use of individual drugs (and derivatively of drug classes).

We showed that when the HIV-RNA covariate is deleted the AUC performance decrease, which is an argument in favour of the use of viral load monitoring for the optimization of cART in LMIC. This reconciles with another recent study from Revell et al. [33], that explored either artificial neural networks or RF for the prediction of virological outcomes in absence of GRT information, using data from Europe, North America, Japan and Australia (>3,000 TCE). Revell et al. showed that, besides the GRT, the viral load is the most important variable, and that TH improves the accuracy of the prediction of virological outcomes.

In a sub-analysis, we showed that the accuracy of the prediction models decreased when training on HIV-1 subtype B data and validating on non-B subtype data. While non-B grouping is an artificial approach not corresponding to any biological entity, differences in response to certain cART regimens with specific subtypes may occur and must be considered in the development of treatment decision tools.

A limitation of our study is that the datasets used do not correspond to a typical scenario of LMIC, since models were applied on data composed by patients cared in Europe. However, we performed sensitivity analyses excluding GRT-guided TCE, and including only first- and second-line regimens with drugs available in LMIC. But a more detailed investigation is advisable since the outcome distributions varied significantly across different datasets. Although the compounds considered in these analyses can be accessible in LMIC, drug combinations might differ.

In addition, in LMIC additional factors like co-morbidity with other diseases, cost of treatment, distance to treatment centres, interruptions of stocks for drugs during certain periods, and stigma can play an important role in treatment outcome. This information needs to be integrated in a system specifically designed for LMIC, provided that co-operation efforts for data collection are appropriately set up.

Another critical point is the fact that therapy switches in EuResist are mainly driven by virological monitoring, and not by clinical/immunological criteria, that are commonly used in LMIC. Finally, in this study a 500-copy threshold in the definition of undetectable viral load was used. This was due to the inclusion of HIV-1 RNA data derived from old generation laboratory assays: although this is a strong limitation for a model designed for high-income countries, where the virological reduction below 50 cp/ml would be the necessary outcome, in LMIC the end-point might be revised by considering the HIV-RNA only where testing is available and CD4/clinical monitoring otherwise.

Despite these limitations, prediction of response to treatment based on TH rather than on GRT appears to be an appealing strategy providing a possibility to help clinicians with data-driven systems in the absence of HIV-1 genotype information. We realize that the here described model is only applicable when therapy failures are mainly judged from viral load monitoring, and when further lines of treatment are available, which is currently not the case in LMIC. However, the concept of the model warrants optimism towards the development of more appropriate models for LMIC. Thus, further development along these line are warranted, along with a coordinated effort to collect HIV-1 treatment related data from the areas that could maximally benefit from it.

## Supporting Information

Material S1 (0.03 MB DOC)

Figure S1 Variable importance evaluation by RF model (ii) on SD8H: mean decrease in Gini index.
(0.17 MB TIF)

Figure S2 ROC plots of a single 10-fold CV run for RF models on SD8H and SD24H excluding instances with an available baseline GRT (n = 9,623) and with/without baseline HIV-RNA load as a covariate.
(0.25 MB TIF)

## References

[1] Thompson MA, Aberg JA, Cahn P, Montaner JS, Rizzardini G (2010). Antiretroviral treatment of adult HIV infection: 2010 recommendations of the International AIDS Society-USA panel.. JAMA.

[2] Gupta RK, Hill A, Sawyer AW, Cozzi-Lepri A, von Wyl V (2009). Virological monitoring and resistance to first-line highly active antiretroviral therapy in adults infected with HIV-1 treated under WHO guidelines: a systematic review and meta-analysis.. Lancet Infect Dis.

[3] Diaz T, Garcia-Calleja JM, Ghys PD, Sabin K (2007). Advances and future directions in HIV surveillance in low- and middle-income countries.. Curr Opin HIV AIDS. 2009 Jul;.

[4] Gupta RK, Pillay D HIV resistance and the developing world.. Int J Antimicrob Agents.

[5] Forster M, Bailey C, Brinkhof MW, Graber C, Boulle A (2008). Electronic medical record systems, data quality and loss to follow-up: survey of antiretroviral therapy programmes in resource-limited settings. Bull World Health Organ..

[6] Gilks CF, Crowley S, Ekpini R, Gove S, Perriens J (2006). The WHO public-health approach to antiretroviral treatment against HIV in resource-limited settings.. Lancet.

[7] Nash D, Katyal M, Brinkhof MW, Keiser O, May M (2008). Long-term immunologic response to antiretroviral therapy in low-income countries: a collaborative analysis of prospective studies.. AIDS.

[8] Walensky RP, Weinstein MC, Yazdanpanah Y, Losina E, Mercincavage LM (2007). HIV drug resistance surveillance for prioritizing treatment in resource-limited settings.. AIDS.

[9] Boyd MA, Cooper DA (2007). Second-line combination antiretroviral therapy in resource-limited settings: facing the challenges through clinical research.. AIDS.

[10] Bishai D, Colchero A, Durack DT (2007). The cost effectiveness of antiretroviral treatment strategies in resource-limited settings.. AIDS.

[11] Moore DM, Mermin J, Awor A, Yip B, Hogg RS (2006). Performance of immunologic responses in predicting viral load suppression: implications for monitoring patients in resource-limited settings.. J Acquir Immune Defic Syndr.

[12] Colebunders R, Moses KR, Laurence J, Shihab HM, Semitala F (2006). A new model to monitor the virological efficacy of antiretroviral treatment in resource-poor countries.. Lancet Infect Dis.

[13] Steegen K, Luchters S, De Cabooter N, Reynaerts J, Mandaliya K (2007). Evaluation of two commercially available alternatives for HIV-1 viral load testing in resource-limited settings.. J Virol Methods.

[14] Marconi A, Balestrieri M, Comastri G, Pulvirenti FR, Gennari W (2009). Evaluation of the Abbott Real-Time HIV-1 quantitative assay with dried blood spot specimens.. Clin Microbiol Infect.

[15] Petrella M, Brenner B, Loemba H, Wainberg MA (2001). HIV drug resistance and implications for the introduction of antiretroviral therapy in resource-poor countries.. Drug Resist Updat.

[16] Beck IA, Crowell C, Kittoe R, Bredell H, Machaba M (2008). Optimization of the oligonucleotide ligation assay, a rapid and inexpensive test for detection of HIV-1 drug resistance mutations, for non-North American variants.. J Acquir Immune Defic Syndr.

[17] Brenner BG (2007). Resistance and viral subtypes: how important are the differences and why do they occur?. Curr Opin HIV AIDS.

[18] Yari A, Passo FS, Hounyet JP (2007). SMARThivPack: A complexity free and cost effective “three tests” combo kit model for improving HIV patients monitoring standards in resource poor settings.. Bioinformation.

[19] Baxter JD, Mayers DL, Wentworth DN, Neaton JD, Hoover ML (2000). A randomized study of antiretroviral management based on plasma genotypic antiretroviral resistance testing in patients failing therapy. CPCRA 046 Study Team for the Terry Beirn Community Programs for Clinical Research on AIDS.. AIDS.

[20] Durant J, Clevenbergh P, Halfon P, Delgiudice P, Porsin S (1999). Drug-resistance genotyping in HIV-1 therapy: the VIRADAPT randomised controlled trial.. Lancet.

[21] De Luca A, Di Giambenedetto S, Cingolani A, Bacarelli A, Ammassari A (2006). Three-year clinical outcomes of resistance genotyping and expert advice: extended follow-up of the Argenta trial.. Antivir Ther.

[22] Alvarez M, García F, Martínez NM, Hernández Quero J, Louwagie J (2004). Retrospective analysis of antiretroviral HIV treatment success based on medical history or guided by the reverse hybridisation LiPA HIV genotyping system.. J Med Virol.

[23] Van Laethem K, Vandamme AM (2006). Interpreting resistance data for HIV-1 therapy management - know the limitations.. AIDS Rev.

[24] Liu TF, Shafer RW (2006). Web resources for HIV type 1 genotypic-resistance test interpretation.. Clin Infect Dis.

[25] Brun-Vézinet F, Costagliola D, Khaled MA, Calvez V, Clavel F (2004). Clinically validated genotype analysis: guiding principles and statistical concerns.. Antivir Ther.

[26] Deforche K, Camacho R, Van Laethem K, Lemey P, Rambaut A (2008). Estimation of an in vivo fitness landscape experienced by HIV-1 under drug selective pressure useful for prediction of drug resistance evolution during treatment.. Bioinformatics.

[27] Altmann A, Beerenwinkel N, Sing T, Savenkov I, Däumer M (2007). Improved prediction of response to antiretroviral combination therapy using the genetic barrier to drug resistance.. Antiviral Ther.

[28] Rosen-Zvi M, Altmann A, Prosperi M, Aharoni E, Neuvirth H (2008). Selecting anti-HIV therapies based on a variety of genomic and clinical factors.. Bioinformatics.

[29] Altmann A, Rosen-Zvi M, Prosperi M, Aharoni E, Neuvirth H (2008). Comparison of classifier fusion methods for predicting response to anti HIV-1 therapy.. PLoS ONE.

[30] Prosperi MCF, Altmann A, Rosen-Zvi M, Aharoni E, Borgulya G (2009). Investigation of Expert Rule Bases, Logistic Regression and Non-Linear Machine Learning Techniques for Predicting Response to Antiretroviral Treatment.. Antiviral Ther.

[31] Larder B, Wang D, Revell A, Montaner J, Harrigan R (2007). The development of Artificial Neural Networks to predict virological Response to combination HIV therapy.. Antivir Ther.

[32] Wang D, Larder B, Revell A, Montaner J, Harrigan R (2009). A comparison of three computational modelling methods for the prediction of virological response to combination HIV therapy.. Artif Intell Med.

[33] Revell AD, Wang D, Harrigan R, Hamers RL, Wensing AM (2010). Modelling response to HIV therapy without a genotype: an argument for viral load monitoring in resource-limited settings.. J Antimicrob Chemother.

[34] Cozzi-Lepri A (2008). Initiatives for developing and comparing genotype interpretation systems: external validation of existing rule-based interpretation systems for abacavir against virological response.. HIV Medicine.

[35] Breiman L (2001). Random Forests.. Machine Learning.

[36] Fawcett T (2006). An introduction to ROC analysis.. Pattern Recognition Letters.

[37] Nadeau C, Bengio Y (2000). Inference for the generalization error.. Advances in Neural Information Processing Systems.

[38] Hastie T, Tibshirani H, Friedman J (2009). The Elements of Statistical Learning.. Data Mining, Inference and Prediction. Second Edition.

[39] R Development Core Team. (2008). R: A language and environment for statistical computing. R Foundation for Statistical Computing, Vienna, Austria.. ISBN.

[40] Witten IH, Frank E (2005). Data mining: practical machine learning tools and techniques..

[41] Altmann A, Däumer M, Beerenwinkel N, Peres Y, Schülter E (2009). Predicting the response to combination antiretroviral therapy: retrospective validation of geno2pheno-THEO on a large clinical database.. J Infect Dis.

[42] Metzner KJ, Giulieri SG, Knoepfel SA, Rauch P, Burgisser P (2009). Minority quasispecies of drug-resistant HIV-1 that lead to early therapy failure in treatment-naive and -adherent patients.. Clin Infect Dis.

[43] Harrigan PR, Wynhoven B, Brumme ZL, Brumme CJ, Sattha B (2005). HIV-1 drug resistance: degree of underestimation by a cross-sectional versus a longitudinal testing approach.. J Infect Dis.

[44] Zaccarelli M, Lorenzini P, Ceccherini-Silberstein F, Tozzi V, Forbici F (2009). Historical resistance profile helps to predict salvage failure.. Antivir Ther.

[45] Le T, Chiarella J, Simen BB, Hanczaruk B, Egholm M (2009). Low-abundance HIV drug-resistant viral variants in treatment-experienced persons correlate with historical antiretroviral use.. PLoS One.

