# Correction: Antiretroviral Therapy Optimisation without Genotype Resistance Testing: A Perspective on Treatment History Based Models

**DOI:** 10.1371/annotation/d0254103-21b9-4078-836b-57ba5bd1c26a

**Published:** 2011-04-26

**Authors:** Mattia C. F. Prosperi, Michal Rosen-Zvi, André Altmann, Maurizio Zazzi, Simona Di Giambenedetto, Rolf Kaiser, Eugen Schülter, Daniel Struck, Peter Sloot, David A. van de Vijver, Anne-Mieke Vandamme, Anders Sönnerborg, for the EuResist and Virolab study groups

Several funding sources were missing from the financial disclosure information. The authors would like to add the following to their funding information: 

"The research team of A-M Vandamme has received funding from the Interuniversity Attraction Poles Programme, Belgian State, Belgian Science Policy (IAP-VI P6/41), from the FWO (grant G.0611.09), and from the AIDS Reference Laboratory of Leuven that receives support from the Belgian Ministry of Social Affairs through a fund within the Health Insurance System.

The European Community's Seventh Framework Programme (FP7/2007-2013) under the project "Collaborative HIV and Anti-HIV Drug Resistance Network (CHAIN)," - grant agreement n° 223131."

