# Supplementary material for: Antiretroviral Therapy Optimisation without Genotype Resistance Testing: A Perspective on Treatment History Based Models
Source: PLoS One. 2010 Oct 29;5(10):e13753. doi: 10.1371/journal.pone.0013753 (PMC2966424; doi:10.1371/journal.pone.0013753)
Supplement: Material S1 — (0.03 MB DOC) [file pone.0013753.s001.doc]

**Supplementary Material**

In figure S1 we depict variable importance calculated by means of 10 independent RF runs on the full SD8H data set. Variable are ranked by mean decrease in Gini index obtained by removing the attribute. In order to take into account potential bias coming from predictor variables that vary in their scale of measurement or their number of categories, distributions were compared with a Student’s t-test (adjusted for multiple comparisons using Benjamini Hochberg method) against others obtained by repeatedly shuffling the class attribute (success at 8-weeks). Values with an adjusted p-value below 0.05 are depicted.

In figure S2 we plot AUC of 10-fold CV for models (ii), (iv) and (v) on the subsets of SD8H and SD24H that do not include GRT-guided TCEs and also by excluding baseline HIV-RNA load as a covariate. TH-based model (ii) significantly outperform models (iv) and (v) at both time points.
