# Supplementary figures and images for: Antiretroviral Therapy Optimisation without Genotype Resistance Testing: A Perspective on Treatment History Based Models
Source: PLoS One. 2010 Oct 29;5(10):e13753. doi: 10.1371/journal.pone.0013753 (PMC2966424; doi:10.1371/journal.pone.0013753)

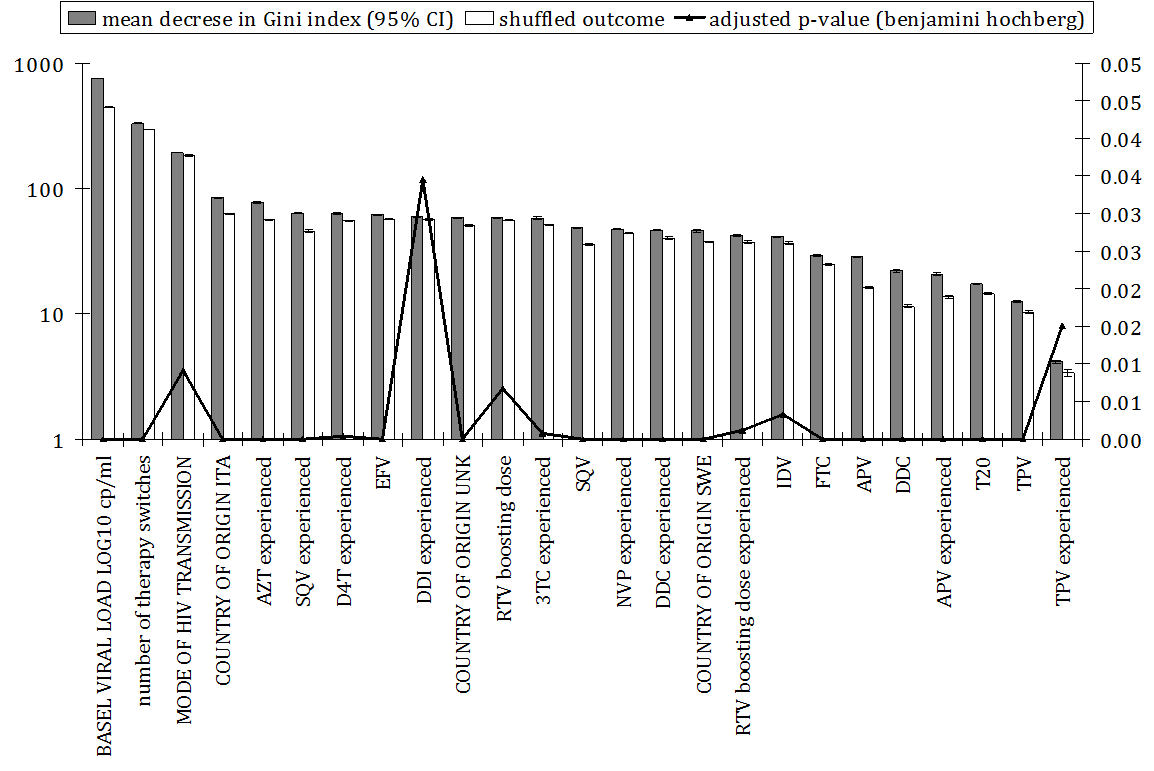

Supplement: Figure S1 — Variable importance evaluation by RF model (ii) on SD8H: mean decrease in Gini index. (0.17 MB TIF) [file pone.0013753.s002.tif]

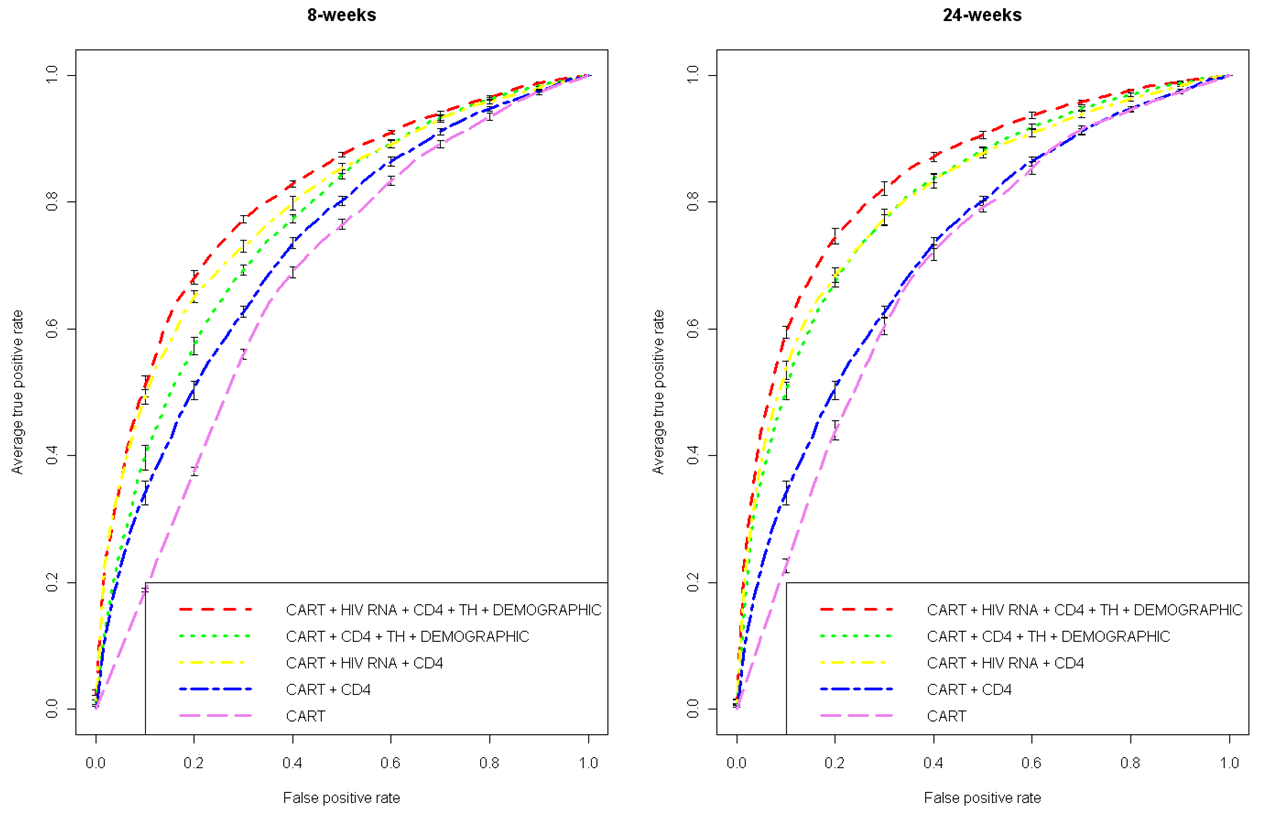

Supplement: Figure S2 — ROC plots of a single 10-fold CV run for RF models on SD8H and SD24H excluding instances with an available baseline GRT (n = 9,623) and with/without baseline HIV-RNA load as a covariate. (0.25 MB TIF) [file pone.0013753.s003.tif]
